# Impact of Transcriptomics on Our Understanding of Pulmonary Fibrosis

**DOI:** 10.3389/fmed.2018.00087

**Published:** 2018-04-04

**Authors:** Milica Vukmirovic, Naftali Kaminski

**Affiliations:** Section of Pulmonary, Critical Care and Sleep Medicine, Precision Pulmonary Medicine Center (P^2^MED), Yale University School of Medicine, New Haven, CT, United States

**Keywords:** interstitial lung diseases, idiopathic pulmonary fibrosis, transcriptomics, biomarkers, microenvironment, microarray, RNAseq

## Abstract

Idiopathic pulmonary fibrosis (IPF) is a lethal fibrotic lung disease characterized by aberrant remodeling of the lung parenchyma with extensive changes to the phenotypes of all lung resident cells. The introduction of transcriptomics, genome scale profiling of thousands of RNA transcripts, caused a significant inversion in IPF research. Instead of generating hypotheses based on animal models of disease, or biological plausibility, with limited validation in humans, investigators were able to generate hypotheses based on unbiased molecular analysis of human samples and then use animal models of disease to test their hypotheses. In this review, we describe the insights made from transcriptomic analysis of human IPF samples. We describe how transcriptomic studies led to identification of novel genes and pathways involved in the human IPF lung such as: matrix metalloproteinases, WNT pathway, epithelial genes, role of microRNAs among others, as well as conceptual insights such as the involvement of developmental pathways and deep shifts in epithelial and fibroblast phenotypes. The impact of lung and transcriptomic studies on disease classification, endotype discovery, and reproducible biomarkers is also described in detail. Despite these impressive achievements, the impact of transcriptomic studies has been limited because they analyzed bulk tissue and did not address the cellular and spatial heterogeneity of the IPF lung. We discuss new emerging technologies and applications, such as single-cell RNAseq and microenvironment analysis that may address cellular and spatial heterogeneity. We end by making the point that most current tissue collections and resources are not amenable to analysis using the novel technologies. To take advantage of the new opportunities, we need new efforts of sample collections, this time focused on access to all the microenvironments and cells in the IPF lung.

## Introduction

**Figure 1:**
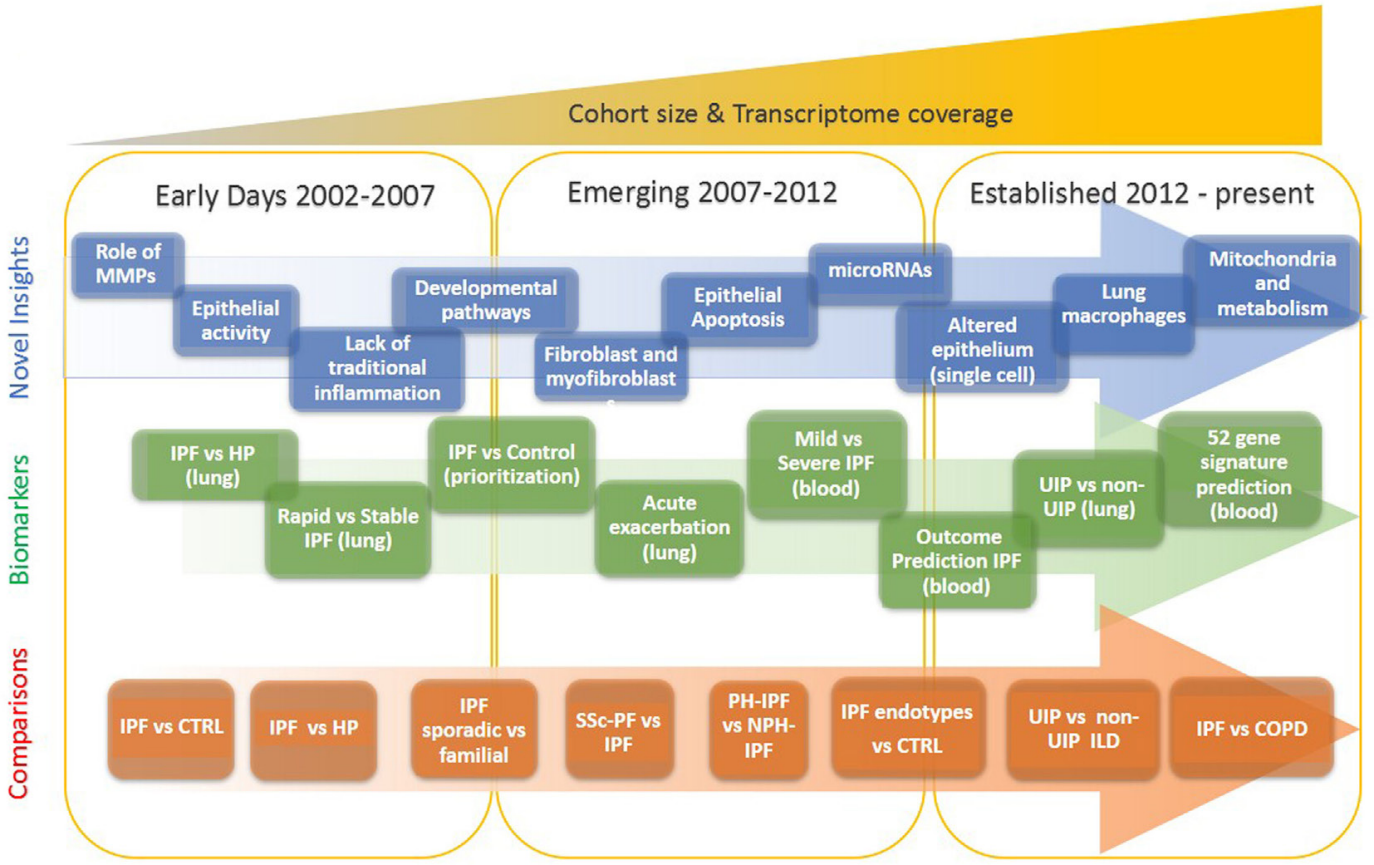
Evolution of idiopathic pulmonary fibrosis (IPF) transcriptome analysis. The progression of IPF transcriptomic research is that of increased complexity, more genes studied, more sample studied, and more detailed phenotypes. In the early days, a few thousand genes were analyzed on a small number of samples and limited analytical approaches. During the emerging period investigators studied tens of samples, mostly on microarrays that profiled of all protein coding mRNAs. In the established period, the numbers of samples are in hundreds, all transcribed RNA is measured, and analytical methods are sophisticated.

Our understanding of idiopathic pulmonary fibrosis (IPF), a chronically progressive scarring lung disease, with a significant genetic component, has dramatically changed in the last two decades. This has happened because after years of formulating hypotheses based on animal models, or analogies from other diseases, pulmonary researchers shifted their focus to analyzing the human lung. The increased availability of well-characterized human tissues and the emergence of high throughput transcriptomic profiling technologies facilitated a new era in IPF research, one in which novel hypotheses are based on observations from human lungs. The sheer size of the data, and its unbiased nature, reintroduced serendipity in pulmonary fibrosis research, and thus led to numerous, previously unexpected observations, novel hypotheses and paradigm shifts. In this perspective, we provide an overview of the impact of transcriptomics on our understanding of IPF. We highlight the timeline of major discoveries (Figure 1) with a focus on mechanisms and pathways, novel biomarkers and disease classification, non-coding RNAs, and disease microenvironments.

## Brief History

The history of transcriptomics in pulmonary fibrosis, is a story of ever increased technological throughput, enhanced sophistication of data analysis and availability of human samples. Gene expression microarrays, which allowed the parallel analysis of hundreds and later thousands of genes, emerged in the second half of the last decade of the twentieth century (1, 2). When the first publication of the application of microarray analysis to pulmonary fibrosis in mice was published in 2000 (3), microarrays could profile ~6,000 transcripts, the statistical approaches were not widely accepted, and human tissues were not available. Two years later, the first analysis of human lungs in 2002 included only eight samples, used a classification algorithm and did not mention a *p*-value (4). These papers were exciting and novel but very limited in numbers of samples and sophistication of analytical approaches.

**Figure 2:**
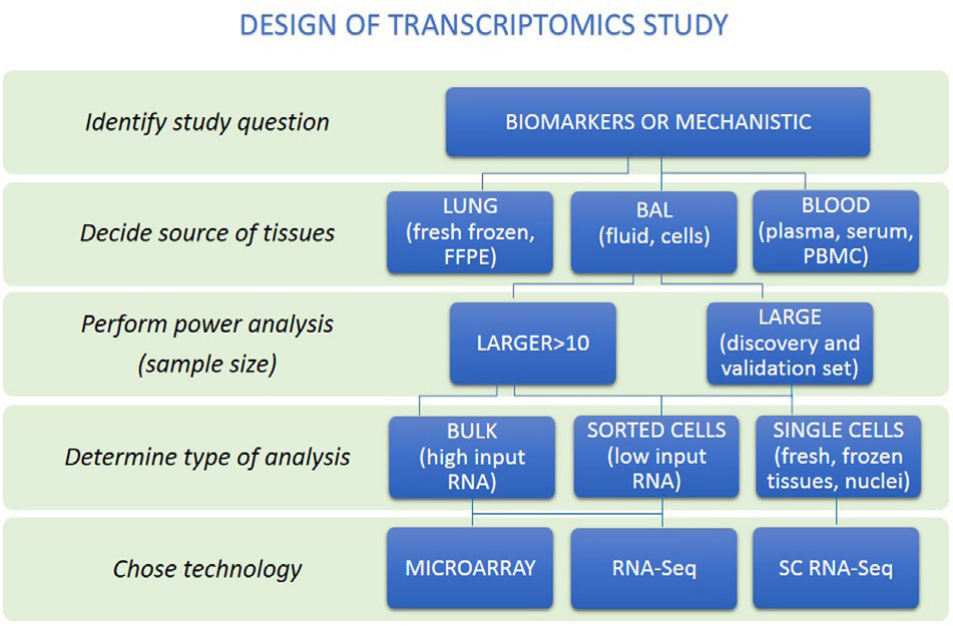
A rational approach to design of transcriptomics study. An overview of steps to help researchers make appropriate study design is presented. First, distinguish whether study aims to identify biomarkers or mechanisms. Then, the source of tissues together with power analysis to calculate sample size to be able to answer research question should be performed. Decision about the type of analysis should be made (bulk, sorted cells, or single cells). Last, the technology to perform transcriptome analysis should be chosen.

Even several years later, studies that aimed at classifying disease included relatively low numbers of samples (5–10). These studies were more sophisticated in data normalization, visualization, and the wide adaptation of statistical approaches to address multiple testing (11, 12). Tissue availability has only increased when NIH-NHLBI established the Lung Tissue Research Consortium, a multicenter publicly available lung tissue repository (13). The expanded availability of tissues allowed application of microarray platforms to hundreds of samples (14, 15) as well the public availability of data through the Lung Genomics Research Consortium (16). Development of RNAseq for deeper sequencing than with microarray platforms resulted in routine profiling of the whole transcriptome including coding and non-coding RNAs, detection of larger dynamic ranges of transcripts, and identification of novel transcripts and variants (17, 18). This further allowed analysis of low-input and degraded RNA samples that enabled research on lung microenvironments and archived tissues (19, 20). Currently, when approaching a transcriptomic study, investigators do not have to be limited by sample or technological feasibility. Instead, they can follow a rational approach to design (Figure 2). The key insights below largely follow aspects of this outline.

## Mechanisms and Pathways

Transcriptomics studies revealed numerous novel molecules and pathways highly relevant for IPF pathogenesis. Here, we describe the most prominent findings, while a more complete list is available in Table 1.

**Table 1:** Summary of relevant idiopathic pulmonary fibrosis (IPF) genes identified by transcriptome profiling.

Gene ID^a^	Gene name	Direction of expression	Tissue localization	Relevant pathway	Reference
**Expressed in lung epithelium in IPF**					
MMP7	Matrix metallopeptidase 7	Increased	Lung (alveolar epithelial cells and fibroblasts), peripheral blood and BAL	Extracellular matrix degradation, defensins, SPP1, and WNT/β-catenin pathway	(4, 5, 21–27)
MMP3	Matrix metallopeptidase 3	Increased	Lung, epithelial cells	Extracellular matrix degradation, β-catenin pathway	(28)
MMP19	Matrix metallopeptidase 19	Increased	Lung, epithelial cells	Extracellular matrix degradation, PTGS2 pathway	(29)
MMP1	Matrix metallopeptidase 1	Increased	Lung, epithelial cells	Extracellular matrix degradation, mitochondrial function/HIF-1-alpha pathway	(30)
SPP1	Osteopontin	Increased	Lung (epithelial cells)	Extracellular matrix degradation	(9, 22)
IGFBP-4	Insulin-like growth factor binding protein 4	Increased	Lung (alveolar and basal cells)	IGF1 pathway	(5, 24)
CCNA2	Cyclin A2	Increased	Lung (alveolar epithelial cells)	Cell cycle regulation	(10)
HIF1A	Hypoxia-inducible factor-1 alpha	Increased	Lung (alveolar epithelial cells)	Hypoxia, p53/VEGF pathways	(31)
CAV1	Caveolin-1	Decreased	Lung	Cell cycle regulation, TGF-b/JNK pathway	(6)
SYN-2	Syndecan-2	Increased	Lung, alveolar macrophages	TGF-b pathway	(32)
TAGLN	Transgelin	Increased	Lung, ATII cells	TGF-b pathway	(33)
CRLF 1	Cytokine receptor-like factor 1	Increased	Lung, ATII	Th1 cells inflammatory response	(34)
EGFR	Epidermal growth factor receptor	Increased	Lung, epithelial cells	Reepithelization	(35)
LYCAT	Lysocardiolipin acyltransferase	Decreased	Lung (epithelial cells), peripheral blood mononuclear cell (PBMC)	Mitochondrial membrane potential	(36)
SERPINF1 (PEDF)	Pigment epithelium-derived factor	Increased	Lung	Angiogenesis	(37)

**Fibroblasts related gene expression in IPF**					
FOXF1	Forkhead box F1	Increased	Lung	COL1/ARPC1 pathway	(38)
VCAM-1	Vascular cell adhesion molecule 1	Increased	Lung, fibroblast foci and blood vessels	TGF-b/ERK/Cyclin D pathway	(39)
FKBP10	FK506-binding protein 10	Increased	Lung, fibroblasts, and CD68 (+) macrophages	TGF-b/Col I synthesis	(40)
RXFP1	Relaxin/insulin-like family peptide receptor 1	Decreased	Lung	TGF-b	(41)
TAZ	Transcriptional coactivator with PDZ-binding motif	Increased	Lung	CTGF and Col1 pathways	(42)
IGFBP3, IGFBP5	Insulin-like growth factor binding proteins 3 and 5	Increased	Lung	IGF pathway	(43)

**WNT pathway in IPF**					
WNT1, 3a, 5a, 7b, 10b, Fzd2 and 3, β-catenin, Lef1, Gsk-3β	Wingless and others	Increased	Lung, fibroblasts, alveolar and bronchial epithelium	Wnt signaling	(23, 44, 45)
LRP5	Wnt co-receptor	Increased	Lung, PBMC	Wnt and TGF-b pathway	(46)
WISP1	Wnt1-inducible signaling protein-1	Increased	Lung	Wnt signaling	(47)

**Apoptotic response in IPF**					
TWIST1	Twist basic helix–loop–helix transcription factor 1	Increased	Lung—fibroblastic foci	Apoptosis/PDGF pathway	(48)
CXCL12	Chemokine ligand 12	Increased	Lung	Inflammation	(8)
TNSF10, BAX, CASP6	Apoptotic regulators	Altered expression	Lung	Apoptosis	(49)
SHP2 (PTPN11)	SH2 domain-containing tyrosine phosphatase-2	Decreased	Lung	Apoptosis/Tyr and Ser/Thr kinase pathways	(50)

**Host defense implicated in IPF**					
DEFA3–4	Defensin alpha 3 and 4	Increased	Lung and peripheral blood	Host defense	(10, 51)
AGER (RAGE)	Advanced glycosylation end product-specific receptor	Decreased	Lung and peripheral blood	Inflammation	(24, 52)

**Mitochondria-related genes in IPF**					
PINK1	PTEN-induced putative kinase 1	Decreased	Lung	Dysfunction of mitochondria	(53)
DIO2	Iodothyronine deiodinase 2	Increased	Lung	TH pathway/mitochondrial biogenesis	(54)

### Matrix Metalloproteinases

Development of IPF was initially explained as fibroblast proliferation, higher expression of tissue inhibitor proteinases (TIMPs), and reduced activity of matrix metalloproteases (MMP) (55). The first study that analyzed human lungs contradicted this paradigm (Figure 1; Table 1). Instead of the expected downregulation, authors found that MMPs were among the most increased genes in IPF lungs including MMP1, MMP2, MMP7, and MMP9 (4). MMP7 was localized predominantly in alveolar epithelium, and MMP7 knockout mice were relatively resistant to fibrosis (4). In addition to MMP7, MMP1 (4), MMP3 (28), MMP19 (29), and MMP28 (56) have been found to be increased in lung epithelial cells of patients with IPF, with diverse and sometimes opposing roles (57, 58).

While their exact roles have not been fully elucidated, the initial unexpected observation that MMPs are increased in the IPF lung, has been validated numerous times. It is now well accepted that MMPs affect numerous signaling pathways that together contribute to the profibrotic environment in the IPF lung and may also serve as effective biomarkers (see below).

### Genes Expressed in Lung Epithelium

Transcriptomic analysis of bulk tissue depends on follow-up analyses to decipher the cellular origin of differentially expressed genes. One of the most surprising findings in IPF transcriptomics was that cellular origin of large number of genes that distinguish the IPF lung from controls ended up being the alveolar epithelium (59, 60) (Figure 1; Table 1). Among the first examples were MMP7, and later SPP1, a protein known to be expressed in inflammatory and bone cells, that in IPF is increased in the epithelial cells adjacent to myofibroblasts foci (22).

Other genes increased in IPF and unexpectedly localized to the alveolar epithelium adjacent to fibrotic regions include N-cadherin (5), HIF-1-alpha (31), IGFBP-4 (9), CCNA2 (10), TAGLN (33), CRLF1 (34), EGFR (35), and DIO2 (54). Among decreased genes, reduced expression of CAV1 (6) and AGER (52) in IPF compared with control lungs was thought to reflect changes in epithelial function or loss of type I alveolar epithelial cells (Table 1).

Of particular interest in this context, is a study that demonstrated that IPF patients with increased expression of cilia genes exhibited also increased MMP7 and MUC5B, as well as microscopic honeycombing but not myofibroblast foci on histological examination, suggesting that they represented a distinct IPF endophenotype (61) (Table 1 and see below).

### Fibroblasts and Fibroblast Foci Related Gene Expression

Genes associated with myofibroblasts, a hallmark of lung histology in IPF, have been described as early as 2002 in bulk tissue analysis (4). Analysis of lung fibroblasts treated with TGFB1 revealed responses to TGFB1 and smooth muscle like myofibroblast phenotype switching (62) that was similar to what was observed in the IPF lung. Fibroblasts isolated from IPF lungs exhibited increased expression of IGFBP3 and IGFBP5 (43), TWIST1 (48), WNT5A (45), COMP (63), and FOXF1 (38). Increased Vascular cell adhesion molecule 1 gene expression in IPF lungs negatively correlated with lung function (39). Another TGFB1 induced gene, FKBP10, a collagen chaperone, was also increased in IPF and IPF lung fibroblasts and contributed to Collagen synthesis (40). Recently, TAZ, a transcriptional coactivator important in development, was shown to be increased in the fibroblastic foci and to contribute to fibrotic response through TAZ-mediated regulation of CTGF (42) (Figure 1; Table 1).

Of particular interest are genes downregulated in IPF lungs and IPF fibroblasts, as they may represent key features lost during disease. RXFP1, a relaxin/insulin-like family peptide receptor is significantly decreased in IPF tissues and fibroblasts and correlates with disease severity. A relaxin-like peptide, CGEN25009 was effective at decreasing bleomycin-induced, fibrosis *in vivo* (41). Similarly, PTPN11, a ubiquitously expressed SH2 domain-containing tyrosine phosphatase, was decreased in IPF lungs and IPF fibroblasts. Overexpression of constitutively active PTPN11 reduced the responsiveness of fibroblasts to profibrotic stimuli, and viral delivery of PTPN11 to wild-type mice blunted bleomycin-induced pulmonary fibrosis (50) (Figure 1; Table 1).

### The WNT Pathway in IPF

Perhaps, one of the most intriguing finding in IPF lungs gene expression was the aberrant activation of developmental pathways and especially the WNT/β-catenin pathway in IPF (Figure 1; Table 1) (64, 65). In 2003, the first observation of β-catenin expression in fibroblastic foci, as well as its expression and colocalization with WNT downstream target genes, CCND1 and MMP7 in adjacent proliferative bronchiolar lesions was reported (64). Subsequently, increased WNT1, WNT7b, WNT10b, FZD2 and FZD3, β-catenin, and LEF1 were found in IPF lungs (23). WNT1, WNT3a, β-catenin, and GSK3B were mainly localized to alveolar and bronchial epithelium with increased expression of WNT targets CCND1 and MMP7. Increased expression of WISP1, a WNT inducible signaling protein, was found in IPF lungs. WISP1 had profibrotic effects *in vitro*, and WISP1 neutralizing antibodies blunted fibrosis *in vivo* (47). Inhibition of WNT/β-catenin pathway attenuated lung fibrosis in mice, suggesting an essential role of WNT/β-catenin pathway in IPF development (46, 66).

While many of these observations were focused on epithelial cells, WNT5A, a member of the non-canonical signaling pathway was increased in IPF lung fibroblasts, with multiple observations suggesting its role in determining fibroblast phenotype in IPF (45, 67, 68).

### Aging, Metabolism, and Mitochondria-Related Molecules

Mitochondrial dysfunction is emerging as one of the key features of IPF. Gene expression data revealed decreased PINK1, a key regulator of mitophagy, and analysis of IPF lungs revealed accumulation of dysfunctional mitochondria in alveolar epithelial cells. Findings from PINK1 knockout confirmed these results, and established a role for impaired mitophagy in IPF (53) potentially through TGFB1 effects (69).

High expression of DIO2, an enzyme that activates thyroid hormone in IPF lungs, and a predisposition to fibrosis among DIO2 knockout mice, led investigators to treat bleomycin treated mice with thyroid hormone or a small molecule agonist (54). Thyroid hormone reversed bleomycin-induced mitochondrial injury both *in vivo* and *in vitro* and augmented resolution of fibrosis in mouse models of pulmonary fibrosis. This effect was dependent on intact PPARGC1A and PINK1 pathways suggesting that the antifibrotic effect of thyroid hormone was mediated through restoration of mitochondrial homeostasis (54).

Changes in expression of genes encoding numerous metabolic enzymes from IPF lungs associated with glucose, fatty acid and citric acid metabolism suggesting on large alterations in mitochondria function (70). Similar findings were found in fibroblasts and alveolar macrophages (71, 72). More detailed review of age-related perturbations in genome and epigenome associating with plausible roles of mitochondria in pathogenesis were published elsewhere (73, 74).

## Gene Expression Patterns as Tools for Disease Diagnosis, Classification, and Outcome Predictors

Transcriptomics studies have also been used to identify disease class related gene expression patterns in the lung, as well as to prioritize protein biomarkers found in the blood stream, or to identify peripheral blood mononuclear cells (PBMCs), gene expression patterns that correlate with disease clinical attributes. The studies are summarized in Table 2.

**Table 2:** Summary of gene signatures that classify interstitial lung diseases.

# Genes	Tissue origin	Disease comparison	Sample size	Year	Reference
407	Lung	Idiopathic pulmonary fibrosis (IPF) vs HP	15 (IPF)12 (HP)	2006	(5)
332/6	Lung	Sporadic IPF vs familial, IPF vs non-specific interstitial pneumonitis (NSIP)	16 sporadic IPF (2 NSIP)10 familial (4 NSIP)	2007	(8)
242/335	Lung, fibroblasts	CTRL vs (SScPF; SScPAH; iPAH; IPF)	33 (15 severe PF, 6 moderate/severe PF and PAH, 4 moderate PF with PAH, 7 PAH), 10 IPF	2011	(75)
<50	Lung	SSc/IFP; IPF vs NSIP	≤10	2007, 2011	(8, 75)
22	Lung	IUP vs (non-IUP, sarc, HP)	77 training set (39 IUP, 38 non-IUP), validation set 48 (22 IUP, 26 non-IUP)	2015	(19)
4,734	Lung	PH-IPF and PAH vs CTRL	18 (PAH), 8 (PH-IPF)	2010	(76)
74	Lung	Chronic lung disease	13 data sets	2015	(77)
>1,500/32^a^	LCM lung	PH-IPF vs CTRL, PH-chronic obstructive pulmonary disease (COPD) vs CTRL, PH-IPF vs PH-COPD	LCM pulmonary arterioles (*n* = 8)	2014	(78)
255	LCM lung	PH-IPF vs NPH-IPF	8 PH-IPF, 8 NPH-IPF	2013	(79)
2,490^b^337^c^214^d^	Lung	IPF vs COPD vs CTRL	19 IPF, 49 COPD	2016	(18)
3 Gene clusters	Lung	IPF vs COPD vs CTRL	319 (3 data sets)^e^	2015	(15)

*^a^32 small DEGs overlap between PH-IPF and PH-COPD*.

*^b^2,490 DEGs between IPF and CTRL*.

*^c^DEGs between COPD and CTRL*.

*^d^DEGs overlap between IPF and COPD*.

*^e^4,259 mRNA and 438 microRNA and also includes 669 clinical variables*.

### Disease Classification

An early suggestion that lung gene expression can be used to classify disease emerged from comparison of lungs of patients with IPF from those with fibrotic hypersensitivity pneumonitis (HP) using transcriptome analysis (5). The enrichment pathway analysis of the HP signature revealed T-cell activation, inflammation, and humoral immune response pathways, whereas the IPF gene signature showed enrichment for cell adhesion, extracellular matrix, and lung development pathways (80).

Analysis of lung samples obtained from patients with sporadic IPF, familial pulmonary fibrosis with a usual interstitial pneumonia (UIP) pattern, and non-specific interstitial pneumonitis (NSIP) revealed similarities on gene expression patterns and pathways and a minimal difference between IPF and NSIP (Table 2) (5, 8). Similar findings were found when systemic sclerosis (SSc) associated pulmonary fibrosis and IPF were compared (75).

A recent study used supervised machine learning algorithms to distinguish lung biopsy samples with UIP from non-UIP (NSIP, sarcoidosis, and HP) identified a 22 gene signature (specificity 92%, sensitivity 64–82%). This approach was solely based on transcriptional data concordant with UIP pathological findings without integration of clinical information, or comparison to patient-level diagnoses by multidisciplinary teams, the current diagnostic gold standard (19). The same group continued improving genomic classifiers to differentiate UIP from non-UIP and demonstrate high robustness toward lung tissue collection using transbronchial biopsy (81, 82) (Figure 1; Table 2).

### Lung Gene Expression Profiles Associated With Disease Activity and Severity

Idiopathic pulmonary fibrosis has different patterns of progression, from stable disease lasting for long periods of time to rapid progression, and acute exacerbations that are highly lethal. Despite a very small number of samples, differentially expressed genes were found in end-stage lungs obtained from patients with rapid and slow progression defined by length of symptoms (Figures 1 and 2; Table 3) (7). Similar findings were also found in a study aimed to identify genes that defined progression by rate of deterioration in pulmonary function tests (9). SFTPA1, SPP1, and HSPA1A were among top increased genes and correlated with worst survival in IPF in agreement with previous reports (83, 84).

**Table 3:** Summary of gene signatures that predict idiopathic pulmonary fibrosis (IPF) progression [rapid vs slow (stable)].

# Genes	Tissue origin	Sample size (IPF)	Year	Reference
437	Lung	26 (rapid progressors), 88 (slow progressors)	2007	(7)
579	Lung	23 (stable), 8 (acute exacerbation)	2009	(10)
134	Lung	6 (stable), 6 (progressive)	2009	(9)
472	Lung	119 (training), 111 (validation)	2013	(61)
468/12^b^	Bleomycin rat/IPF human	100 (human), 73 (rats)	2015	(14)
1,428/2,790/13^a^	Peripheral blood mononuclear cell (PBMC)	130 (mild vs ctrl; severe vs ctrl; mild vs severe)	2012	(51)
118	PBMC	45 (training), 21 and 75 (validation)	2015	(85)
52	PBMC	45 (discovery), 75 (validation), and 425 (validation)	2013, 2017	(86, 87)

*^a^13 DEGs between mild and severe IPF*.

*^b^12 is set of translational markers*.

The study of acute exacerbations of IPF has been limited, because of lack of tissue availability. Using a unique resource of rapid lung autopsies (88) investigators compared lung gene expression profiles of acute exacerbations, stable end-stage IPF, and controls (10). They did not find any significant evidence for infection or overt inflammation in acute exacerbation lungs, but they did find increased expression of CCNA2, and DEFA3 and DEFA4, antimicrobial proteins of the alpha-defensin family known to be cleaved by MMP7 (25) and evidence for widespread epithelial apoptosis.

A more sophisticated effort to identify disease endotypes based on tissue gene expression, incorporated clinical and histological information in the analysis (61). This determined that patients with increased expression of cilia-related genes, such as DNAH6, DNAH7, DNAI1, and RPGRIP1L, exhibited also increased expression of SPP1, MMP1, MMP7, PLUNC, MUC5B, as well as more microscopic honeycombing on histology but no myofibroblastic foci (61) (Table 3). Interestingly, MMP7 has previously been shown to attenuate ciliated cell differentiation during wound repair (27). Another effort to identify disease activity genes studied gene expression commonalities between IPF disease progression in humans and bleomycin-induced lung fibrosis in rats (14). They identified the largest overlap in differentially expressed genes between lung transcriptome of bleomycin-induced fibrosis and IPF human lungs and identified 12 genes (C6, CTHRC1, CTSE, FHL2, GAL, GREM1, LCN2, MMP7, NELL1, PCSK1, PLA2G2A, and SLC2A5) as translational markers of disease activity. Of those markers, four classified IPF patients based on disease severity (14).

### Cross Disease Endotypes

The availability of large datasets such as the LGRC, allowed also analysis of multiple chronic lung disease in parallel. Recently, applying a novel computational approach named integrative phenotyping framework, investigators discovered novel endotypes of chronic obstructive pulmonary disease (COPD) and IPF (15). They integrated clinical phenotype data with mRNA and microRNA data and identified novel patient clusters. The genes that characterized the patients in the intermediate clusters were enriched with inflammatory and immune pathways, suggesting that patients from those clusters could have a mechanistically distinct autoimmune endotypes (15). Similarly, the same group integrated mRNA, microRNA, and splicing gene variants to identify convergent transcriptional regulatory networks in IPF and COPD (18). The p53/hypoxia pathway emerged as a convergent pathway in COPD and IPF. A recent study performed meta-analysis of 13 published data sets including cystic fibrosis, COPD, IPF and asthma, environmental conditions (smoking, epithelial injury), and control to identify general markers of chronic lung disease (77). Increased inflammatory, wounding, defense response and regulation of cell proliferation pathways, and decreased immune response pathways were observed (77). While intriguing, all of these studies were limited by lack of resolution with regard to cellular admixture and depth of clinical phenotyping (Figure 1; Table 2).

### Prioritization of Protein Biomarkers

Genome scale transcriptome studies facilitated the development of protein-based biomarkers for IPF diagnosis (Figures 1 and 2; Table 4). A comparison of proteins in the blood flow of patients with IPF to control using a targeted proteomic approach identified a signature of MMP1, MMP7, MMP8, IGFBP1, and TNFRSA1F (24) that was able to distinguish IPF from controls with high sensitivity and specificity. MMP 1 and MMP 7 were also increased in the lungs of IPF patients and able to differentiate IPF patients from other chronic lung disease including hypersensitivity pneumonitis and sarcoidosis.

**Table 4:** Summary of single genes—biomarkers of idiopathic pulmonary fibrosis (IPF) progression.

Gene ID	Gene name	Tissue origin	Sample size (IPF)	Year	Reference
MMP7	Matrix metallopeptidase 7	Lung, serum, plasma, BAL	13 (lung), 74 (plasma, lung, BAL)20 (BAL)214 (plasma, 140 derivation and 101 validation)65 (serum), 1,227 (serum), 97 (plasma)	2002, 2008200920122016, 2017	(4, 24)(89)(26)(90, 91, 92)
SPP1	Osteopontin	Lung, BAL	18	2005	(22)
COMP	Cartilage oligomeric matrix protein	Lung	115	2013	(63)
CXCL13	C–X–C motif chemokine 13	Lung, plasma	92, 94	2014	(93)
CCL8	Chemokine (C–C motif) ligand 8	Lung, BAL, plasma	8 (lung), 86 (BAL, plasma)	2017	(94)

Indeed, MMP7, which emerged out of the first microarray analysis of human IPF lungs, was replicated as predictive of increased mortality in multiple cohorts of IPF patients (14, 26, 90–92, 95). Similar experimental strategy, following a lung gene expression finding with assessment of a protein in the peripheral blood, has been applied to many molecules including SPP1 (22), COMP (63), CXCL13 (93), CCL8 (94), and others (Table 4).

### Peripheral Blood Gene Expression Patterns

The transcriptome of the peripheral blood is highly appealing because of information about disease presence and outcome. It represents a safe and accessible alternative to availability of samples from the lung. Microarray gene expression profiles of whole blood RNA (51) distinguished IPF patients from controls, and among IPF patients, 13 genes were changed with increased disease severity as assessed by DLCO but not FVC (Figures 1 and 2; Table 3) (51). Interestingly, alpha-defensins identified in acute exacerbations in the lung (10) were also associated with disease severity in the peripheral blood.

A subsequent study aimed to identify PBMC gene expression profiles predictive of increased mortality in patients with IPF (86). The authors performed microarray analysis on RNA isolated from PBMCs in discovery and replication cohorts of IPF patients. They identified a 52-gene outcome-predictive signature that distinguished two patient groups with significant differences in transplant free survival in both cohorts. Interestingly, increased mortality was associated with decreases in the T-cell co-stimulatory molecules CD28, ICOS, LCK, and ITK, potentially highlighting the role of potential T-cell aberrations and maybe the role of immunosenescence in IPF. Remarkably, the outcome-predictive accuracy of a score calculated based on the 52-gene signature was recently validated in a six cohorts study containing 425 IPF patients (87). Adding the 52-gene risk score to the Gender, Age, and Physiology index significantly improved its mortality predictive accuracy, suggesting that the genomic signature improved on the performance of validated clinical markers. Analysis of longitudinal changes in the signature revealed that while the 52-gene risk score tracked changes in FVC, patients never shifted their risk profile. However, in a subset of treated patients, a shift in the risk score was also accompanied by functional improvement, suggesting that the 52-gene signature may be indicative of response to the therapy. These datasets were also used in manuscripts that applied Weighted Gene Co-expression Network Analysis to identify gene expression modules that correlate with outcome (85) or microbiome changes (96) (Table 3). The impressive accuracy and replication should drive experiments that test the value of these biomarkers prospectively and assess in detail shift in circulating inflammatory cells in IPF using unbiased methods such as single-cell RNAseq.

## Role of Non-Coding RNAs in IPF

Until recently considered the dark matter of the genome, the significant role of non-coding RNAs in human health and disease is increasingly appreciated (97). We will focus here on microRNAs, as their role has been extensively studied in pulmonary fibrosis.

### MicroRNA Changes Reveal Loss of Differentiation

MicroRNAs are small non-coding RNAs that regulate gene expression by either initiating RNA degradation or inhibiting translation through binding to the 3′ UTR of their target gene. Acting as rheostats, many microRNAs regulate the general responsiveness of a cell to a certain stimulus by affecting numerous genes and frequently serving as gate keepers of feed forward loops. The expression of approximately 10% of the microRNAs is different in IPF compared with control lungs (98, 99). The microRNA expression patterns observed in IPF are similar to those observed in the developing lung. Comparison of fetal, IPF and control lungs revealed that miR-487b, miR-409-3p, miR-154, miR-154*, miR-134, miR-299–5p, miR-410, miR-382, miR-377, and miR-296 were increased in IPF or fetal lungs compared with controls (99). A time course systems biology analysis of microRNAs changed during postnatal lung development suggested that close to 40% were also changed in IPF (100). In the same vein, comparison of microRNA signatures in IPF and non-small cell lung cancer revealed significant similarities and numerous microRNAs that changed in the same direction. Notably, over 20 microRNAs including members of the miR-30, let-7, miR-29 families were decreased in IPF and lung cancer, commonly increased microRNAs included miR-155, miR-21, miR-205, and miR-31 (101). While the cellular origin and exact effects of all of these common microRNA changes are unclear, together with the observations about lung development, microRNA changes in the IPF lung suggest a loss of the differentiated organ regulatory networks potentially as a result of desynchronized aging (102, 103).

### IPF MicroRNAs and TGFB1

One of the most recurrent themes in microRNAs in IPF, is that they are both regulated by and regulators of TGFB1 signaling. Thus, in many cases, a change in the expression of a microRNA disrupts the careful balance of self-limited activation of TGFB1. Let-7d, a microRNA known to regulate epithelial cell differentiation, is a good example. It is decreased in IPF lungs, it is inhibited by TGFB1 through direct effect of SMAD3, and when it is inhibited, it ceases to inhibit HMGA2, allowing amplification of TGFB1 signaling and early fibrotic changes *in vivo* and *in vitro* (98). Similarly, miR-21, a microRNA increased in IPF lungs, is induced by TGFB1 and is an inhibitor of SMAD7, a regulatory SMA that inhibits TGFB1 signaling pathways (104). A larger number of TGFB1 inducible microRNAs, localized to chromosome 14q32, were also increased in IPF lungs (99). Other microRNAs regulating or regulated by TGFB1 were found to be changed in IPF lungs include miR-30, miR-199, miR-29, miR-26, miR-155, miR-326, and others (105). While, it can be safely said that microRNA changes in IPF seem to result in lowering the cell profibrotic threshold, it has to be mentioned that they were obtained in isolation, for one microRNA at a time, but in the IPF lung, at least when analyzed in bulk, they happen simultaneously. To understand better the effects of microRNA perturbations, careful dissection of the cellular, spatial, and temporal changes, as well as their integrated effects is required.

### miR-29, the Ultimate Antifibromir

Of microRNAs differentially expressed in IPF, the miR-29 family is probably the most extensively studied both mechanistically and as a therapeutic target, because of its known inhibitory effects on extracellular matrix proteins, and growth factors such CTGF and IGF1 (106). miR-29 family members are decreased in cardiac, renal and liver fibrosis, keloid, fibrotic Crohn’s disease, and other fibrotic conditions (107–113). miR-29 family microRNAs are decreased in IPF lungs (114), they regulate numerous genes related to fibrosis (115) and seem to regulate profibrotic signals from the extracellular matrix to fibroblasts (116). Both gene delivery of miR-29 *via* a transposon method (117) or using a miR-29b mimic (118) augmented resolution of bleomycin-induced pulmonary fibrosis. While most of these studies focused on the role of miR-29 in fibroblasts, two recent studies suggested that miR-29 could be important in prevention of pulmonary fibrosis (119) or bronchopulmonary dysplasia (120) through beneficial effects on alveolar repair. Regardless of the cell specificity of the effect, miR-29 supplementation seems a viable option as an antifibrotic therapy.

## IPF Microenvironments

The IPF lung is characterized histologically by its regional, temporal and cellular heterogeneity, meaning that normal looking regions are interspersed with diseased regions, different regions may appear at different stages of disease (121, 122), and both the cellular content and the phenotype of known cells are dramatically altered in the IPF lung. Transcriptomic profiles of bulk tissue homogenates do not capture this complexity. They also do not allow understanding how cells influence each other in the remodeled IPF microenvironment. Improving the cellular and spatial resolution of transcriptomics using single cells and tissue microenvironments is critically important to decipher what happens in the IPF lung.

### Tissue and Cellular Heterogeneity Are Starting to Emerge

Transcriptome analyses performed on bulk lung tissue detected strong gene expression signals, leading to discovery of IPF relevant signaling pathways (Figures 1 and 2). However, it is unclear whether alteration in transcriptome signals represented core features of disease or was dominated by changes in cellular admixture. Increased gene expression changes observed in the IPF lung were frequently assigned to cell types, based on prior knowledge or follow-up studies, as in the case of MMP7, SPP1, WISP1, COMP, TWIST1, PINK1, and the others mentioned earlier. In most cases, such analysis was done after the fact, using low throughput technologies such as immunohistochemistry, and was dependent on prior knowledge and availability of reagents. Only few studies analyzed transcriptomic gene expression in well-defined IPF microenvironments. Comparison of the transcriptome of hyperplastic vs conserved epithelial cells and dense fibrotic lung regions, using laser capture microdissections identified previously unrecognized MMP19, as a molecule increased in hyperplastic epithelial cells, with an antifibrotic role (29). Two studies reported solely gene expression profiles of pulmonary vasculature and showed differential gene expression for IPF patients with and without coexistent PH (79) and for PH-IPF and COPD (78) (Table 2). Two clusters of co-regulated genes related to bronchiolar epithelium or lymphoid aggregates were identified when whole lung transcriptome was correlated with tissues histology and clinical variables (123). The first study to apply single-cell RNAseq of sorted epithelial cells from IPF patients or controls revealed distinct epithelial cell types in IPF lung and complete lack of some “normal” epithelial cells (124). Using transcriptomic profiling of flow-sorted cells, monocytes shown to differentiate into alveolar macrophages and continuously express profibrotic genes over the course of fibrosis. Thus, selective targeting of alveolar macrophage differentiation within the lung may decrease fibrosis and avoid global monocyte or tissue-resident alveolar macrophage depletion (125). Besides transcriptomics profiling of sorted and single cells isolated from fresh lung, the RNAseq of archival formalin-fixed paraffin-embedded lung biopsy from IPF patients is possible (20). This allows analysis of specific areas of lungs and their interaction observed microscopically (epithelium and fibroblastic foci), usage of clinical variables (survival) and overcoming the availability of fresh lung tissues.

While lung microenvironment studies are still rare, the rapid emergence of methods for high throughput sequencing of single cells, the improved ability to perform sequencing from IPF microenvironments, the improved analytical methods, and the success of old fashioned analyses of bulk tissue should encourage investigators to perform larger studies focusing on understanding temporo-spatial multicellular networks in IPF.

## Conclusion and Future Directions

**Figure 3:**
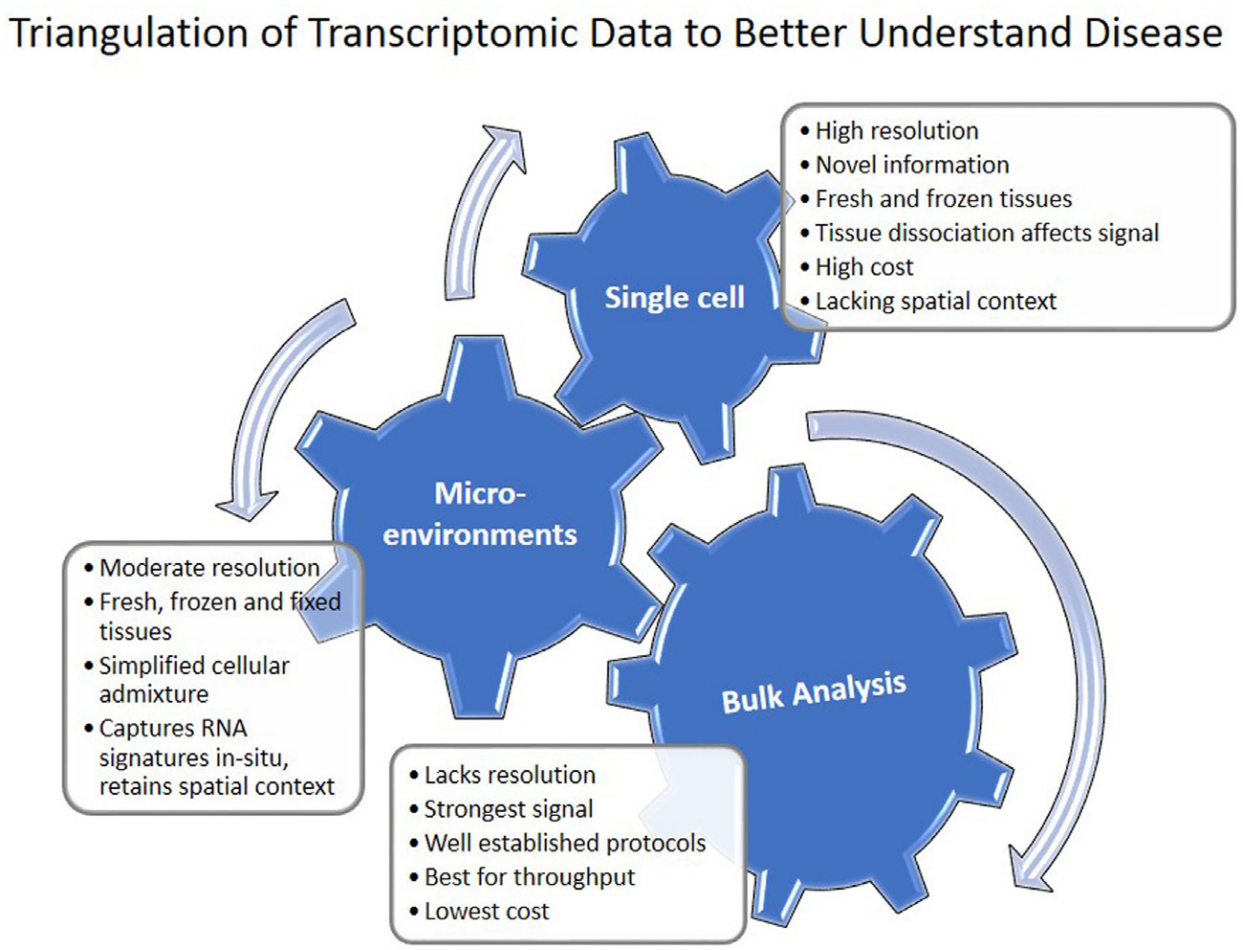
Triangulation of transcriptomic data to understand disease. Single cell, microenvironment, and bulk tissue transcriptomic analysis have their advantages and disadvantages. When applied together, they can help in understanding regulatory networks in the tissue.

The progress of transcriptomics in IPF is characterized by increased sophistication and complexity (Figure 1). Transcriptomics studies facilitated multiple shifts with regard to the role of MMPs, developmental pathways, microRNAs, and the importance of alveolar epithelial and myofibroblast regulatory networks in IPF. They have also had significant impact on the discovery and prioritization of validated biomarkers (Figure 1). However, most of these studies used low sample number and lack validation cohorts. NIH NHLBI funded efforts led to generation of publicly available datasets of multi-omics data generated from carefully characterized human and mouse samples (Table 5). They contain, mainly bulk tissue, but also limited amounts of sorted cells and single-cell transcriptomic profiles. With the advent of novel technologies for single cell and microenvironment transcriptomic profiling, we have a unique opportunity to triangulate IPF regulatory and transcriptional networks by analyzing the lung from a verity of perspectives, use available bulk data, as well as profiles of disease microenvironments and single cells (Figure 3). This will allow integration of information and resolution of the cellular, temporal, and spatial complexities of the IPF lungs and thus better therapeutics and diagnostics. In 2014 following a series of meetings sponsored by NIH-NHLBI, the Pulmonary Fibrosis Foundation and the American Thoracic Society Assembly of Respiratory, Cell and Molecular Biology convened a series of meetings that recommended among other things, an open access biorepository for IPF research (126). While various registries have been formed, new centralized efforts to obtain IPF lung tissues have not been renewed. This is a problem, because most current tissue collections are not amenable to analysis using the novel technologies. To take advantage of the new opportunities, to continue the momentum of transcriptomic success we need new efforts of sample collections, this time focused on access to all the microenvironments and cells in the IPF lung.

**Table 5:** Data and tissue repositories.

Name	Website	Reference
Lung Tissue Research Consortium	http://www.ltrcpublic.com/	(126)
Lung Genomics Research Consortium	http://www.lung-genomics.org/	(126)
Lung development map	https://www.lungmap.net/	(127, 128)
Cell differentiation analysis (scRNAseq)	http://www.cs.cmu.edu/~jund/scdiff/index.html	(129)

## Author Contributions

MV and NK substantially contributed to review design; data analysis and interpretation. Both the authors participated in writing and revising the review, approved the final work, and agreed to be accountable for all aspects of the review.

## Conflict of Interest Statement

NK is an inventor on a pending patent on use of thyroid hormone as an antifibrotic agent (licensed), as well as a patent on novel biomarkers in IPF (not licensed). NK consulted Biogen Idec, Boehringer Ingelheim, Numedii, MMI, Pliant, Third Rock, and Samumed. NK has an ongoing collaboration with MiRagen but no fund exchange. The other author declares that the research was conducted in the absence of any commercial or financial relationships that could be construed as a potential conflict of interest.
